# Details on the transport of European eel larvae through the Strait of Gibraltar into the Mediterranean Sea

**DOI:** 10.1038/s41598-024-82929-z

**Published:** 2025-01-06

**Authors:** Marko Freese, Lasse Marohn, Luis Ferrer, Jan-Dag Pohlmann, Klaus Wysujack, Tina Blancke, Reinhold Hanel

**Affiliations:** 1 Thünen Institute of Fisheries Ecology, Federal Research Institute for Rural Areas, Forestry and Fisheries, 27572 Bremerhaven, Germany; 2 AZTI, Marine Research, Basque Research and Technology Alliance (BRTA), Herrera Kaia, Portualdea z/g, 20110 Pasaia, Spain

**Keywords:** *Anguilla anguilla*, Recruitment, Leptocephalus, Tidal cycles, Internal waves, Animal migration, Marine biology, Physical oceanography, Animal behaviour

## Abstract

Numbers of European glass eels (*Anguilla anguilla)* monitored along the Atlantic and Mediterranean coasts of Europe currently serve as the main stock indicator in assessment of this critically endangered species. Spawning, however, takes place exclusively in the Sargasso Sea, several thousand kilometers away. The beginning of its complex lifecycle is characterized by a distant and lengthy larval drift, before the young-of-the-year reach the monitoring stations at the European coasts. The oceanic mechanisms regulating dispersal and distribution of European eel leptocephalus larvae, before they metamorphose into glass eels and colonize future growth habitats, are still poorly understood and data are scarce. Here, we present oceanographic and leptocephalus catch data from a 24-h station on board of the German Research Vessel Meteor, covering one event cycle of the tide-derived change of hydrographic conditions in the central part of the Strait of Gibraltar. Results of this study provide detailed insights on how the exchange of water masses between the Atlantic and the Mediterranean Sea may favor or prevent transport and migration of eel larvae through the Strait, which potentially plays a decisive role in timing and magnitude of larval recruitment events into the entire Mediterranean region.

## Introduction

The stock of the panmictic European eel (*Anguilla anguilla*) exhibited substantial decline in recent decades and is currently rated as critically endangered by the IUCN Red List of Threatened Species^1^. Recruitment time-series for glass eels and yellow eels, monitored along the Atlantic, Baltic and Mediterranean coastlines, currently serve as the basis for the evaluation of the status and development of the stock^2^. While these data provide crucial insights into recruitment dynamics, they do not capture all potential threats or changes affecting the whole stock, emphasizing the need for a comprehensive understanding of the complete life cycle including the leptocephalus stage^3–5^. Especially early life history stages such as eggs and larvae are vulnerable to a number of additional oceanic and environmental factors^6–12^. Investigating and surveying changes in oceanic abundance and distribution of larvae can be valuable to define the role of oceanic factors in the species decline and provide information on the temporal and regional fluctuations in coastal recruitment^7,13^. Further, knowledge about the transport and dispersal mechanisms of leptocephalus larvae in the open North-West Atlantic, but also in the coastal areas after their journey from the Sargasso Sea, are key to understand and predict the magnitude of yearly recruitment in different areas^14^. Ocean currents play a decisive role in timing as well as trajectory of the drifting leptocephalus larvae, as distinct current patterns have been identified that either favor a transport to the North Sea or towards the Mediterranean Sea^4,11,15–18^.

Besides oceanographic features, larval behavior can also be a key element in this regard since anguillid leptocephali are not seen as fully passive drifters and observations as well as behavioral studies have shown their ability to actively swim horizontally and to rise and dive in the water column^19–21^. Even though availability of empiric data on the vertical distribution of leptocephalus larvae in the water column is not extensive, current understanding is that larger, later stage anguillid leptocephali conduct diurnal vertical migrations (DVM) and are usually found in deeper water layers during daytime but in shallower, surface-near layers during the night^13,20,22,23^. Previous studies described the preferred depth during nighttime to range between 50 and 70 m, while the extent of vertical distribution during daylight seems to increase with body length^24^. Tesch^23^ reported large leptocephali close to the European shelf to reside mainly between 300 and 600 m during daytime. Previous studies have shown that this distinct behavior also mirrors in the catchability of the larvae in certain depth ranges, which is the reason why the sampling strategies and targeted depth strata for specimens of this life history stage usually differ during night and day^22,25^. The vertical position of the larvae during different times of day and night thus potentially affects their oceanic distribution, as direction and velocity of currents often vary depending on the depth stratum. Not only due to the complexity of larval oceanic dispersal and the wide natural distribution, which increase the efforts to develop robust monitoring strategies, there remains a paucity of data on recruitment and standing stock in certain parts of the eel’s range. Glass eel monitoring at selected sites in the Mediterranean and close to the Strait (e.g. at the Guadalquivir River, located on the Spanish Atlantic side) are considered crucial data points to describe recruitment trends in the Mediterranean Sea^26^. Still, the Mediterranean Sea is particularly considered inadequately covered by recruitment time-series, even though the area is thought to be an important habitat for the species. Until today, it is neither entirely clear in what magnitude its production contributes to the spawning stock biomass, nor precisely how high the natural recruitment is in this area^2,13,26–28^.

The Mediterranean Sea and the Atlantic Ocean are connected by the Strait of Gibraltar, a narrow (∼13 km at its narrowest point) and rather shallow (∼280 m at the shallowest point, the Camarinal Sill) bottleneck, which young eels inevitably need to pass in order to colonize any coastal area or river system of the Mediterranean or Black Sea. This, of course, is also true for adult silver eels emigrating out of the Mediterranean, in order to successfully conduct their spawning migration^24,27,29^. Similar to other gateways between different ocean basins, water exchange, current and tidal systems in the Strait of Gibraltar are complex and regulated by a variety of factors^24^. Semi-enclosed basins, such as the Mediterranean, typically display differences in several physical characteristics from the open Atlantic Ocean^29^. The water balance of the Baltic Sea for instance, is controlled by outflow of a freshwater and precipitation-fed brackish water column within the upper layer^30^, while inflow is characterized by high saline water from the Kattegat in deeper strata^31^. In contrast, the Mediterranean Sea is considered a concentration basin, which is characterized by water loss due to evaporation exceeding water gain from precipitation and river runoff. Adding to this, there is a negative net heat budget of the basin, causing an anti-estuarine vertical thermohaline circulation, as deeper, more saline water exits the Mediterranean, while surface waters from the Atlantic enter the Mediterranean^32,33^. Additionally, these density-driven currents are superimposed by tidal currents that can exceed two meters per second and thus strengthen or slow down the eastwards-directed (i.e., towards the east sector, including east, east-northeast, and east-southeast directions) surface inflow, depending on the phase of the tide.

The tidal energetics of the Strait of Gibraltar are complicated and have been intensely studied^33–37^. However, even though notable scientific effort in collecting data for this region was made, for a long time, the tidal regime of the Strait was considered still not entirely understood by some authors^38^. The current flow is assumed to interact with the Camarinal Sill (shallow part of the Strait) causing surface wave patterns, that are detectable by satellites^35,39^. The fluctuating conditions and temporarily strong currents in the Strait in connection with the limited mobility of the few centimeters long leptocephali suggest that recruitment and transport from the Atlantic into the Mediterranean must be linked to specific conditions, as was already supposed by Tesch et al. in 1986^40^. The significance of the area for the distribution of eel larvae was also highlighted in a dedicated paragraph in McCleave et al.^24^, who hypothesized that the tidal conditions in the Strait make a passive transport of relatively shallow residing leptocephali during spring tide not only possible, but likely. In more detail, and considering the behavioral characteristics of eel larvae (DVM) as well as the oceanographic conditions in the Strait, it was hypothesized that optimal conditions for the larval transport into the Mediterranean Sea would only be given, when strong eastward surface currents occur at nighttime after sunset, when the eel larvae ascend to the upper part of the water column. The here presented study builds on this work by providing oceanographic and larval catch data during a 24-h station to complement the hypothesis with concrete data. The aim of this study was therefore to gain better insight into the transport of anguillid leptocephali at a highly dynamic bottleneck during their transoceanic larval migration. Results are based on periodical plankton sampling with an Isaacs-Kidd Midwater Trawl (IKMT) in the central part of the Strait of Gibraltar. During a full tidal cycle, we monitored velocity in the water column using acoustic doppler current profiler (ADCP), additional hydrographic parameters using a conductivity, temperature and depth rosette (CTD) as well as surface current velocity and direction derived from high-frequency radar (HFR).

## Results

**Fig. 1:**
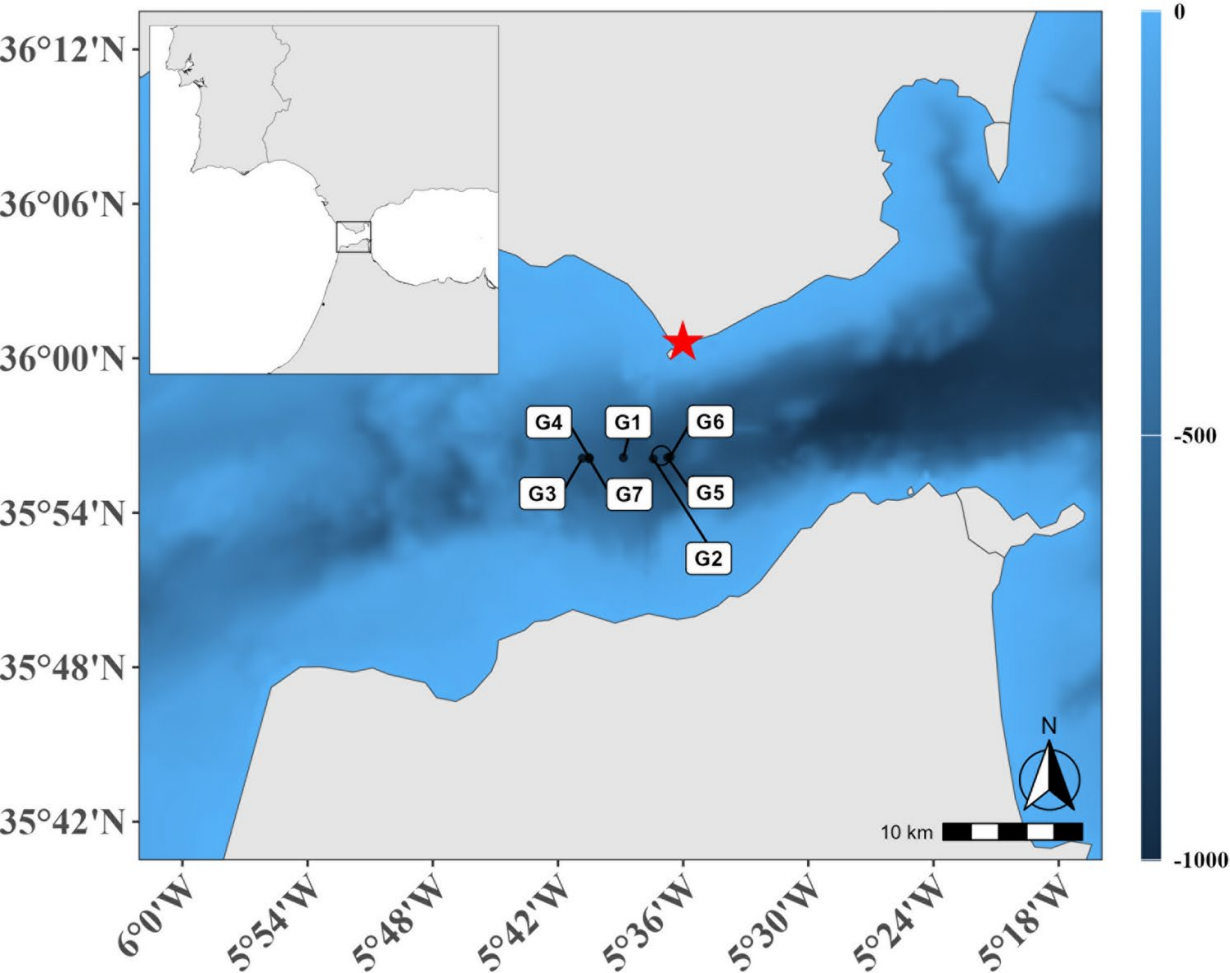
Map of the Strait of Gibraltar (including an overview map in the top left corner) depicting the starting (black circle) and ending positions (black dots with haul-ID) for each haul of the IKMT during the 24-h sampling station as well as bathymetric contours. The position of the tidal gauge (https://portus.puertos.es/*)* is indicated by a red asterisk. Both the detailed map and the overview map were generated using R (version 4.4.1, https://www.r-project.org) with the giscoR and marmap packages, based on data from NOAA (https://nauticalcharts.noaa.gov/data/data-licensing.html). The overview map was integrated into the detailed map using Photoshop Elements 2021 (version 19.0, https://www.adobe.com/products/photoshop-elements.html).

Seven deployments of the IKMT during 24 h in the central part of the Strait of Gibraltar (Fig. 1) revealed clear differences in leptocephalus catch quantities. While zero larvae were caught during the two daytime and one evening hauls in depths from 0 down to 200 m, a total of 31 leptocephali including 17 *Anguilla anguilla* (total length (TL): 54–65 mm), 12 *Conger conger* (TL: 50–71 mm), 1 *Eurypharynx pelecanoides* (TL: 16 mm) and 1 *Gnathophis* sp. (TL: NA) specimen were caught in the upper 100 m during the four nighttime hauls. A total of 13 *A. anguilla* and 4 other leptocephali were caught during one (G5) of the deployments on November 21st beginning at 01:00 UTC. This haul produced the highest catch per unit effort (CPUE) of 9.51 caught larvae per hour, as well as the highest calculated larval density (3.31*10^−4^ individuals / m^3^) in this study and the entire survey^41^ .

### Weather and oceanic conditions

Wind direction and speed (Table 1) during the observed time frame were moderate and tidal current velocities can be considered common for the Strait at this time of the year. The tides on November 20th and 21st, 2022 occurred during a waning moon, meaning that sampling did not take place during extreme tidal ranges like those seen during spring tides (Table 2). Higher windspeeds and/or spring tide conditions would have had an increasing effect on the currents in the Strait.

**Tabl﻿e 1:** Station name, date and starting time, starting position, maximum catch depth as well as catch metrics including catch per unit effort (CPUE) of each IKMT haul conducted during the 24 h station in the middle of the Strait of Gibraltar.

Haulname	Station	Date/time (UTC)	Latitude (N)	Longitude (W)	Max. depth (m)	*Anguilla* (N)	Other leptos (N)	Catch time (min)	Filtered volume (m^3^)	CPUE (N/hour)	*Anguilla* density (10^–4^ N / m^3^)	Day/night (d/n)	Average wind velocity (m s^−1^)	Wind direction (°)
G1	M185_41-3	20.11.2022 08:42	35°56.119	5°36.790	200	0	0	59	14,099	0	0	d	6.36	300
G2	M185_41-7	20.11.2022 16:19	35°56.219	5°36.808	200	0	0	70	26,290	0	0	d	8.06	275
G3	M185_41-8	20.11.2022 19:05	35°56.156	5°37.154	200	2	2	74	24,104	1.62	0.83	n*	6.53	285
G4	M185_41-9	20.11.2022 21:12	35°56.150	5°37.350	100	2	1	56	16,692	2.14	1.20	n	5.89	294
G5	M185_41-11	21.11.2022 01:00	35°56.092	5°36.758	100	13	4	82	39,231	9.51	3.31	n	6.13	278
G6	M185_41-12	21.11.2022 02:41	35°56.107	5°36.737	100	0	7	88	42,287	0	0	n	7.06	268
G7	M185_41-14	21.11.2022 07:02	35°56.090	5°36.971	100	0	0	92	40,709	0	0	n/d	9.17	226

Note that haul G3 was deployed during early evening and towed deeper than the other night hauls and that G7 was conducted in early morning during dawn, which is why this haul cannot be classified as a pure day or night haul.

**Table 2:** Tides from the Tarifa tide gauge (36.01º N, 5.6º W) and wind speeds from the meteorological station Dique del Puerto de Tarifa during the ADCP sampling in the Strait of Gibraltar.

Tide	Date (day/month/year) and time (UTC)	Height (m)	Average wind velocity (m s^−1^)
Low	20/11/2022 04:32	−0.31	5.1
High	20/11/2022 11:03	0.44	6.2
Low	20/11/2022 17:11	−0.39	7.6
High	20/11/2022 23:31	0.45	3.4
Low	21/11/2022 05:07	−0.38	3.6
High	21/11/2022 11:44	0.52	8.5
Low	21/11/2022 17:40	−0.48	14.3

The height is referenced to the local mean sea level (source: Puertos del Estado, https://portus.puertos.es/).

### Water stratification in observed timeframe

**Fig. 2:**
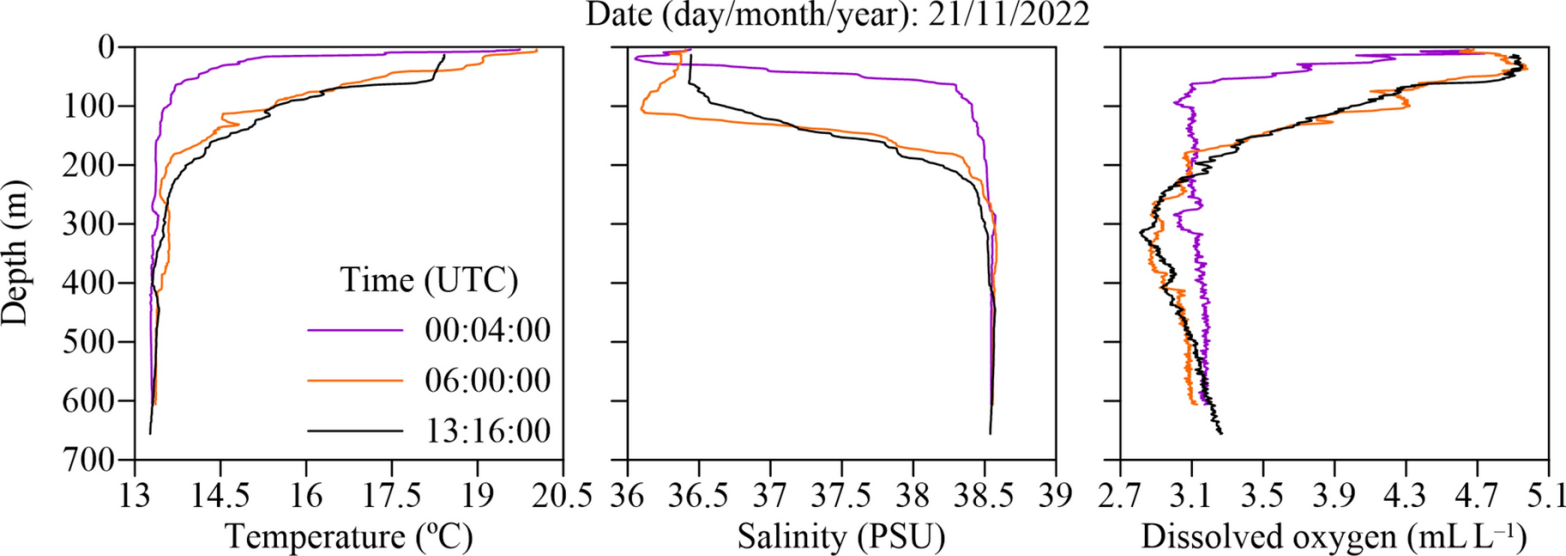
CTD profiles of a stratified water column shown by temperature (°C), salinity (PSU) and dissolved oxygen (mL L^−1^) taken in the central part of the Strait of Gibraltar on November 21st, 2022, at 00:04, 06:00 and 13:16 UTC.

**Fig. 3:**
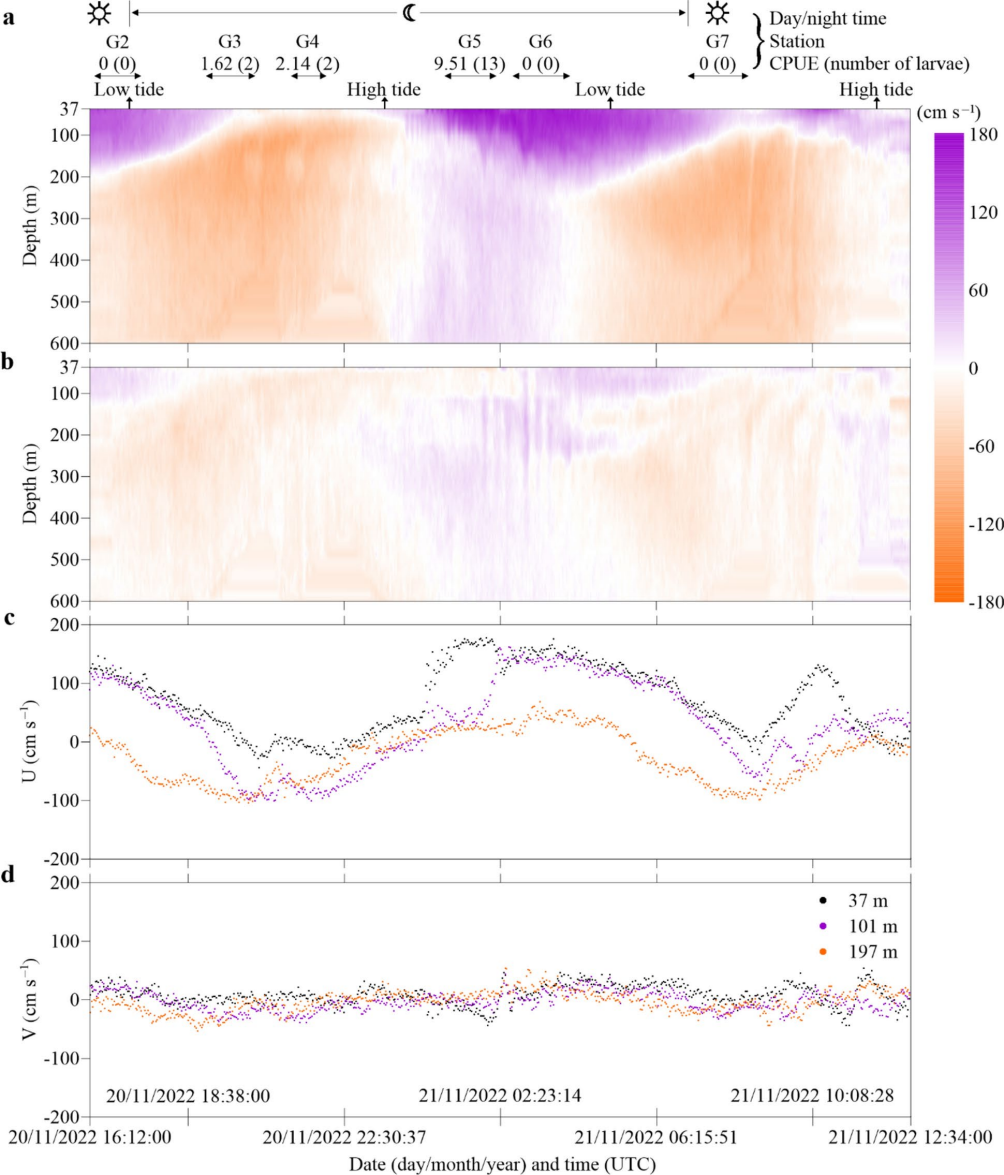
Shipboard Acoustic Doppler Current Profiler (ADCP) velocity measurements in the Strait of Gibraltar during cruise M185. (**a**,** b**) Vertical sections of the eastward (a) and northward (b) velocity components. (**c, d**) Eastward (U) and northward (V) components at three depths (37, 101 and 197 m), respectively. Day and night phases, haul ID, catch numbers as well as low and high tides at the Tarifa tide gauge (36.01° N, 5.6° W, source: Puertos del Estado) are indicated above.

CTD data (Fig. 2) confirmed a stratified water column in the converging area in the central part of the Strait of Gibraltar, which corresponded well with the known separation of water masses from the Atlantic and the Mediterranean Sea. While the surface layer was characterized by a 70–100 m deep lens of warmer, less saline and less dense water, the deeper layers of the water column were characterized by more saline, colder and thus denser water masses. In addition to these characteristics, higher oxygen levels in the surface layer indicate a stronger mixing in this depth stratum. The CTD profiles at 00:04, 06:00 and 13:16 UTC (Fig. 2) reflect the tidal influences on the water stratification (Fig. 3). The profile at 00:04 (purple) was recorded shortly after high tide and showed lower temperatures and higher salinity between 37 and 250 m depth due to the westward transport of Mediterranean water. In contrast, the profile at 06:00 (orange) was recorded after low tide, with the influence of eastward flowing Atlantic water leading to higher temperatures and lower salinity in the same depth range. The profile at 13:16 (black), taken after the second high tide, was consistent with the observed westward transport below 300 m. These fluctuations, which are supported by ADCP measurements (Fig. 3), illustrate the influence of tidal cycles on the distribution of water masses.

### Water current velocity and direction measured by acoustic Doppler current profiler (ADCP)

For technical reasons, the shipboard ADCP velocity measurements started with haul G2 in the afternoon of November 20th and (in correspondence with CTD data) also showed a stratified current structure in the water column. Strong variation was present in the eastward velocity component (Fig. 3a,c) that flowed from the open Atlantic towards the Mediterranean Basin and rather negligible variation in the northward velocity components (Fig. 3b,d). For the eastward component, current velocities generally differed during the observed timeframe depending on depth, with highest velocities in the upper 100 m, reaching a maximum of almost 200 cm s^−1^ due to strong inflow from the Atlantic into the Mediterranean (Fig. 3a). High velocities of more than 120 cm s^−1^ were observed at the end of the first monitored outgoing tidal cycle (20/11/2022, approx. 16:20 UTC) until shortly after the first low tide (20/11/2022, approx. 16:50 UTC), as well as between the first high tide until after the second low tide of the observed timeframe (21/11/2023, approx. 00:15–08:00 UTC). This is highlighted in Fig. 3c for the exemplary depths 37 m (black) and 101 m (violet), starting around 00:00 UTC on November 21st until after low tide, approx. 08:00 UTC on November 21st, which was shortly after the last haul (G7). Deeper water strata, exemplified here by the depth of 197 m (orange), show a negative eastward movement (i.e., a westward movement) during most of the tidal cycle, and neutral or only very slight eastward movement occurring between high tide and low tide (Fig. 3a,c).

**Fig. 4:**
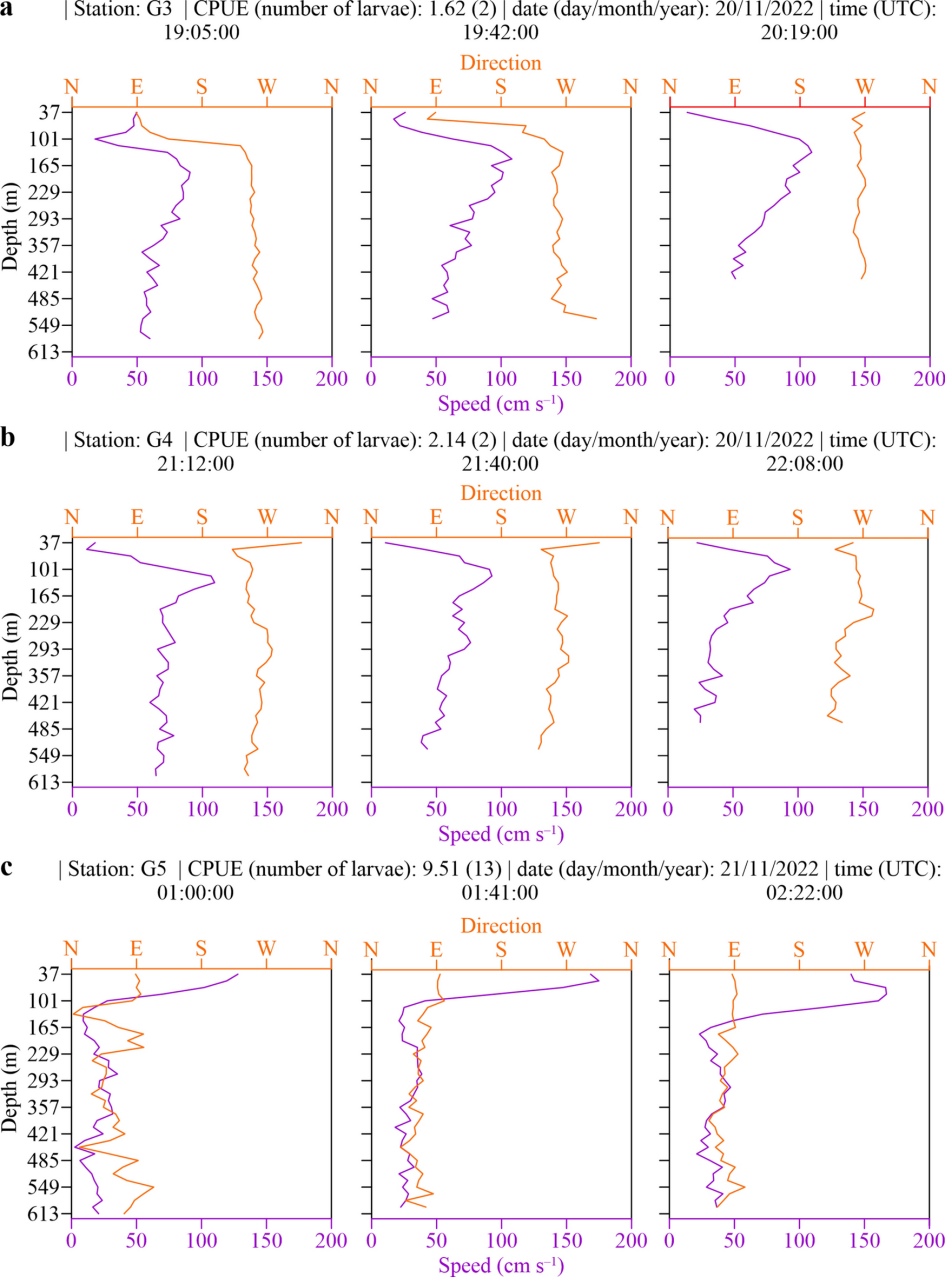
ADCP-derived water velocity magnitude and current direction at three times (beginning, mid-haul and end of haul) during stations with leptocephalus catches. N, E, S, W directions correspond to 0º, 90º, 180º and 270º, respectively.

ADCP vertical profiles of water velocity magnitude and direction during the timing of the most successful haul (G5) depict the magnitude and direction of the inflow event in eastward direction (Figs. 3, 4c). Water profile plots of current speed and direction at three exemplary times (beginning, mid-haul, end of haul) of hauls with caught anguillid larvae (G3 (n = 2), G4 (n = 2) and G5 (n = 13)) further depict the respective prevailing current regimes in different depths (Fig. 4a–c). While current direction and speed during the first haul with caught larvae in the upper water layer (37–100 m) in the beginning and mid haul was directed eastward to southward at a moderate velocity ranging from 25 to 50 cm s^−1^ (Fig. 4a), the water turned directions amidst the haul completely westwards through the entire water column from surface down to 600 m (Fig. 4a). The second haul with caught larvae (Fig. 4b) displayed a westward direction in the very upper water layer and a south-western trajectory in the beginning and mid haul. Current speed was slow to moderate, ranging from approx. 10–76 cm s^−1^, with peaks around 100 cm s^−1^ between 100 and 165 m of depth. The highest observed current velocities were seen in G5, the haul with the highest count of collected anguillid leptocephali (Fig. 4c). During the three exemplary times of this haul, current velocity reached 175 cm s^−1^ in the upper 100 m of the water column in an eastward direction. Current direction in greater depths during this haul turned from north-eastwards to eastwards at low to moderate speed ranging from 0 to 40 cm s^−1^ (Fig. 4c).

### Atlantic jet sea surface current derived from high-frequency radar

**Fig. 5:**
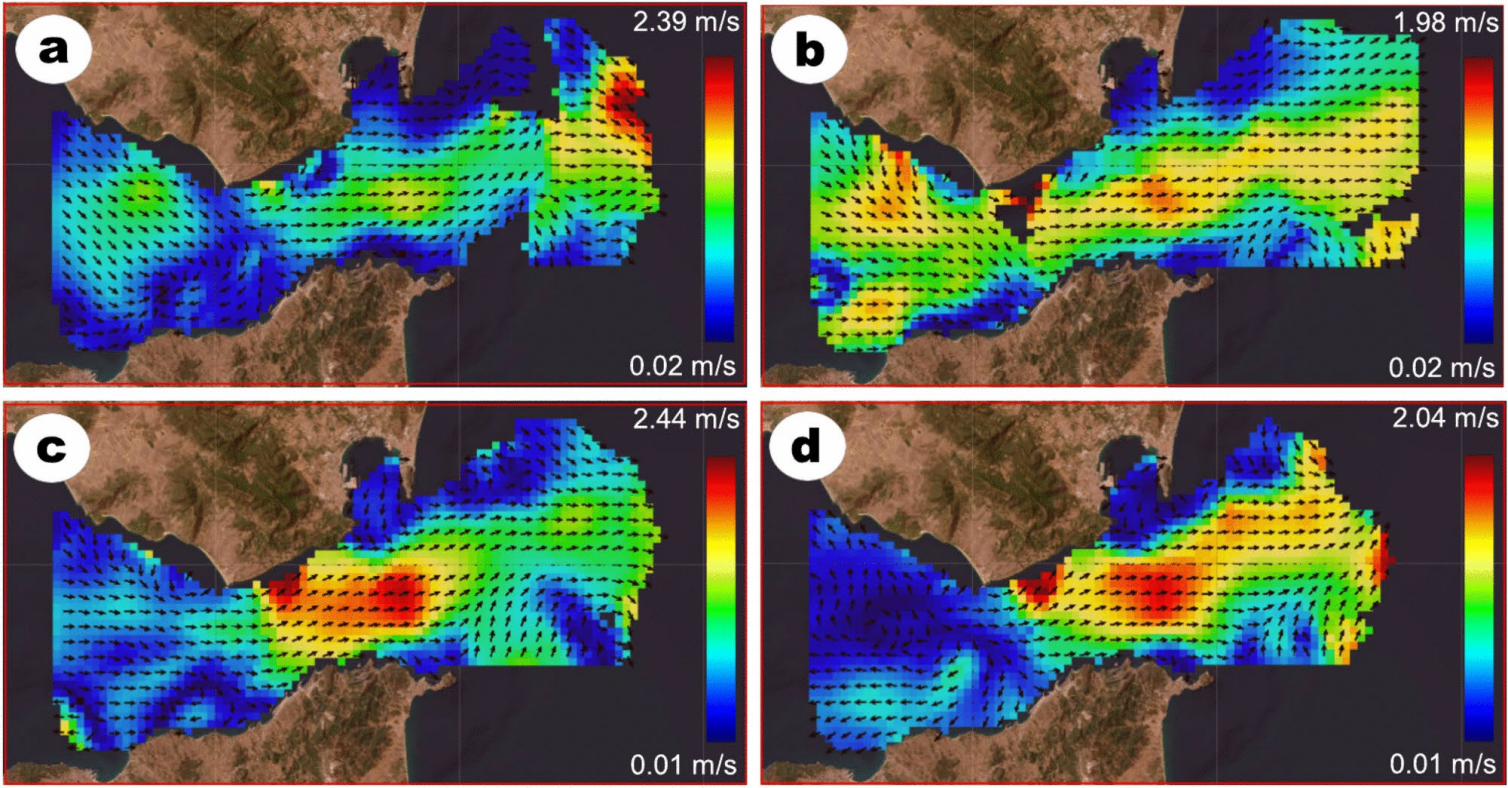
Direction (arrows) and speed (colors) of sea surface currents on November 20th, 2022 at 23:00 UTC (a, top left) and on November 21st, 2022 at 02:00 UTC (b, top right), 05:00 UTC (c, bottom left) and 08:00 UTC (d, bottom right) measured by the Spanish high-frequency radar in the Strait of Gibraltar. The used available range of the radar is displayed here as colored vectors, ranges out of limits are displayed in black. (HFR domain: 35.78°–36.22° N, 4.86°–5.93° W, source: Puertos del Estado, https://portuscopia.puertos.es/).

Data derived from high-frequency radar monitoring sea surface currents in the Strait of Gibraltar show a strong inflow event from November 20th, 2022 at 23:00 UTC until November 21st, 2022 at 08:00 UTC (Fig. 5). High eastward velocities (yellow & red) characterized the Atlantic jet current, which moved surface water masses eastwards through the Strait of Gibraltar from the Atlantic into the Mediterranean (Fig. 5c,d).

## Discussion

This study is the first to provide catch data and environmental data as connected evidence of how eel larvae are transported from the Atlantic to the Mediterranean Sea. We provide further support for the hypothesis by McCleave et al.^24^ that optimal conditions for larval transport into the Mediterranean Sea arise when strong eastward surface currents occur at night, coinciding with the ascent of eel larvae into the upper, shallower layers of the water column as part of their diel vertical migration behavior. Utilizing this ‘perfect wave’ of strong eastward currents appears to facilitate the most effective transport of larvae. However, it is important to note that the necessary conditions are not uniform throughout the year. In winter, southern Spain experiences longer periods of darkness (nearly 14 h) compared to summer. This extended darkness aligns with tidal cycles, providing more potential windows for larval eel transport than in summer. The exact timing of larval transport within the water column, and whether it occurs right after sunset or a later at night, may affect their interaction with tidal conditions. If larvae migrate upwards shortly after sunset and return to deeper waters at sunrise, winter conditions with longer nights could offer more favorable conditions for transport compared to summer, especially during spring tides or strong westerly winds. Miller et al.^4^ published a comprehensive review on the distribution, timing and size of *A. anguilla* leptocephali, documenting that late stage leptocephali have been collected almost throughout the entire year around the Strait of Gibraltar. It is unclear if the abundance of leptocephalus larvae in this area greatly changes throughout the year, or if there is a potential peak of arrival at a specific timeframe. But while it seems reasonable to assume that there might be a temporal connection to a time before the peak arrival of glass eels in estuaries around the Strait and in the Mediterranean, it remains unclear if and to what extent larval abundances in this area change throughout the year.

Relating the ADCP and HFR data with the IKMT catches of this study, larvae were only collected at nighttime and highest leptocephalus abundance (G5) was recorded during the formation of an eastward directed wave of Atlantic water towards the Mediterranean (Figs. 2, 4c). The other two hauls (G3 and G4) containing *Anguilla* larvae were conducted during non or only slowly westward flowing incoming tide, as can be seen in Fig. 4. These larvae were possibly in the surface strata of a water mass that has not been entirely pushed through the Strait but instead was (at least in parts) pulled back to the western Atlantic part of the Strait when the tides changed. The outflow of the heavier, more saline water out of the Mediterranean Sea through the Strait in westward direction possibly constitutes a natural barrier for leptocephalus larvae in front of the Strait. Miller^20^ underlined how poorly documented the swimming capabilities of leptocephalus larvae were. Even though it cannot be ruled out that anguillid eel larvae are capable of single-direction swimming to increase recruitment success^19^, their capability of actively swimming long distances or against strong currents has been subject to scientific discussion and speculation in the past^4,15,24,40^. Only few publications provide empiric data on the swimming capacity of anguillid larvae: Artificially hatched 30–60 mm long larvae of the Japanese eel (*Anguilla japonica*) reached swimming speeds of 3.6 + /- 2.7 cm s^−1^ horizontally and 2.8 + /- 1.1 cm s^−1^ vertically in captivity, which corresponds roughly to one body length s^−1^^21^. Interestingly, this constitutes a swimming speed that has shown the best hypothetical recruitment success in models, compared to inactive drifting^42^. Swimming speeds of late stage or metamorphosing *Conger oceanicus* leptocephali and *A. rostrata* glass eels, measured in a laboratory swimming channel, were faster: ranging from 12.0 to 26.8 cm s^−1^ and 4.1 to 25.0 cm s^−1^, respectively^43^.

During the 24 h observation period, current velocities in deeper, westward flowing water layers ranged from approx. 50–100 cm s^−1^, except during the tidal jet surface inflow associated with Atlantic high tides. These current velocities, compared to the known swimming speeds of larvae, suggest that active swimming of anguillid leptocephali against the outflowing currents of the Mediterranean water during daytime over a longer time span and distance must be deemed unrealistic. Tesch et al.^40^ were apparently the first to address this question after a larval survey in the area and described a stowing or bottleneck effect in the funnel between Europe and Africa, just before the Strait of Gibraltar. The authors also concluded that a swimming speed of at least 20 cm s^−1^ was required to effectively compensate westwards outflowing currents from the Mediterranean. Our data support this, as the observed tidal dynamics, which are characterized by fast eastward-flowing surface water strata and stationary or slowly westwards-moving deeper layers could explain accumulation effects of leptocephali west of the Strait. These accumulations likely persist until the next eastward tidal pulse transports the larvae during their shallow nighttime phase of the DVM.

Current direction and velocity in the Strait of Gibraltar are greatly influenced by the Atlantic tidal system, resulting in a constant move of oceanic water from the Atlantic into the Mediterranean and back. Our recordings during the 24 h station in the Strait are consistent with a model that describes the exchange of water masses between the oceanic bodies as tidal oscillation waves with movement patterns in different directions, yet mainly following an eastward trajectory^44^. Izquierdo et al.^45^ considered tides to be by far the most energetic process in the Strait, that present a semidiurnal character, though diurnal current velocities were not negligible. These tides are essential for the water movement in the Strait all year round, so although there was not much wind on this occasion, the observed conditions during the 24 h station can be regarded as representative of average conditions in the Strait of Gibraltar. While most of the time heavier, more saline water pushes out in deeper layers westwards of the Mediterranean Sea into the Atlantic, high tides and rising water levels in the Atlantic facilitate intensification of eastward surface currents, pushing into the Strait (e.g. as can be seen in the eastward directed wave in Figs. 2c, 4a–d) with velocities of close to 200 cm s^−1^ (e.g. 7.2 km h^−1^ or > 20 km in three hours tidal phase) and more in the upper 50–100 m of the water column. As during these conditions, also the deeper water layers move eastwards (Fig. 4c), this (albeit considerably slower) could enable recruitment into the Mediterranean also in greater depths during daytime, when the larvae still linger in deeper waters.

Given that the European eel is a panmictic species, a selective and directed recruitment into the Mediterranean Sea seems unlikely. Yet, the mechanisms, timing and triggers that initiate leptocephalus larvae to metamorphose into glass eels and colonize continental habitats remain poorly understood. While for glass eels, it was shown that a geomagnetic sense could guide them to use tidal cycles and orientate towards the coastal shores to colonize coastal and freshwater habitats^46^, it is unknown whether eel leptocephali have similar capabilities to guide their transoceanic migration^47,48^. Regardless of this, larval as well as metamorphosing eels exhibit negative phototaxis, as demonstrated for captive reared Japanese eel larvae, for which overhead light caused clear downward movement in experimental works^21^. In the same artificially spawned larvae, Yamada et al.^21^ observed that swimming speeds during horizontal movement and rising behavior were significantly higher than during diving behavior and suggested that this may help to regulate distribution during inshore migration of young arriving recruits. Tidal waves pushing surface water through the Strait into the Mediterranean Sea during nighttime could therefore be regarded as a transport pump that will directly affect dispersal of eel larvae and other organisms conducting nocturnal vertical migrations into the surface strata of the water column. Macias et al.^34^, who studied the influence of internal waves on the composition and abundance of zooplankton in the Strait, already highlighted the need to understand and discuss the physical components and processes when addressing biological patterns in regions with intense hydrodynamic regimes.

Despite some limitations due to the confined timeframe of this study, the obtained data and observations are consistent with expectations and the hypotheses presented herewith. Further research covering longer timeframes ideally throughout other times of the year together with depth-stratified sampling would help to test the here presented hypotheses. Future studies should include consideration of peak arrival of recruits in the area, environmental factors such as weather conditions, different tidal phases and changing environmental conditions caused by climate change.

## Conclusions

This study describes how the interaction between diurnal vertical migration behavior of leptocephalus larvae and the tidal conditions in the Strait of Gibraltar likely enables the recruitment of European eel larvae into the Mediterranean Sea. The presented data suggest that a significant eastward surface current in combination with the ascent of leptocephali into the upper water layers at nighttime effectively transports larvae from the Atlantic Ocean to the Mediterranean Sea via the Strait of Gibraltar. Consequently, the here presented data contribute to our knowledge about the hydrographic factors determining the magnitude and timing of early life stage recruitment events into the Mediterranean and could help develop convenient targeted and effective recruitment monitoring strategies for this area, complementary to glass eel recruitment time-series.

## Methods

The collection of oceanic data, current parameters, as well as the sampling of leptocephalus larvae, was conducted in November 2022 during one leg of the 185th cruise (M185) aboard the German Research Vessel METEOR.

### Leptocephalus catch data

All leptocephali in this study were collected using a 1550 µm mesh-size Isaacs-Kidd Midwater Trawl (IKMT) with a ~ 7 m^2^ mouth opening and a length of 12 m (HYDRO-BIOS Apparatebau GmbH, Altenholz, Germany). Seven hauls were conducted during a 24-h station between the 20th and 21st of November 2022 in the middle of the Strait of Gibraltar, located roughly at 35°56 N and 5°37 W (Fig. 1; Table 1). The ships speed over ground was adapted to current speed in order to facilitate the gear’s ideal and comparable trawling speed of 2.5 knots in water. As a result, starting and ending points but also the timing of vertical positions in each haul varied (See Fig. 1 & S1 for details).

Filtered water volumes were assessed with flowmeters (2030R, General Oceanics, Miami, USA). As stratified sampling was not possible during this cruise, the IKMT was towed in three double oblique hauls between surface and 200 m of depth during daytime and early evenings and in four quadruple oblique hauls between surface and 100 m of depth during nighttime and early morning, to target only surface-near leptocephali after or during their diel vertical migration (See Table 1 & S1 for details). This was done to be able to appoint the caught larvae to the upper water strata. Fishing at deeper strata would have potentially captured deeper larvae and thus may have led to misinterpretation of larval vertical distribution. Collected larvae were immediately sorted from the catch, morphologically identified, measured, tissue-sampled for genetic species determination and then stored at −20 ℃ for further investigations. All leptocephalus larvae sampled at this station were genetically confirmed by DNA barcoding.

### Genetic species confirmation

Tissue samples from each leptocephalus larva were separately preserved in ethanol (96% abs.) on board for subsequent genetic analysis. After the survey, DNA was extracted using Chelex100^49^ and stored at 4 °C for several weeks or at −80 °C for long-term storage. The verification of morphologically identified Anguillid species was carried out by a Real-Time PCR-based assay, detecting *A. anguilla* and *A. rostrata*in comparison to reference samples. All remaining leptocephalus larvae were morphologically identified, with their species identity confirmed through Nucleotide BLAST^50^ analysis of the mitochondrial DNA barcoding marker gene Cytochrome c oxidase I (COI), which was amplified by PCR and sequenced using Sanger sequencing (Service lab: StarSEQ GmbH, Mainz, Germany).

### Oceanographic data

As a standard procedure, prior to every deployment of the IKMT during the 24-h station, vertical observations of temperature (℃), salinity (PSU), water density (kg m^−3^) and oxygen (mL L^−1^) were collected by using a SBE 911 conductivity temperature-depth measure (CTD) (Sea-Bird Electronic, Bellevue, WA, USA). All CTD casts were conducted from sea surface to 600 m, respectively.

### ADCP data

Ocean current velocities (from approx. 37 m to the sea floor, depending on the region and sea state) were collected along the cruise track by a vessel-mounted Teledyne RD Instruments 38 kHz Ocean Surveyor ADCP (Acoustic Doppler Current Profiler). The shipboard ADCP provides estimates of the horizontal velocity components as a function of depth, based on the Doppler effect of sound wave reflections against small particles carried by ocean currents. The transducer was located at 5 m below the water line, mounted in the hull of the ship. The instrument was operated in narrowband mode with 16 m bins and a blanking distance of 16 m, while 100 bins were recorded using a pulse of 2.89 s. The measurements obtained were averaged over 2 min. For more details, see Hanel et al.^51^. The maximum depth reached in the Strait of Gibraltar was approx. 626 m.

### High-frequency radar

High-frequency radar (HFR) data was derived from in situ instruments operated by Puertos del Estado in the Strait of Gibraltar. The HFR system deployed in Strait of Gibraltar consists of four sites equipped with a CODAR-Seasonde model. The sites, owned and operated by Puertos del Estado, started data collection in May 2011. The system works at a central frequency of 26.8 MHz, providing quality-controlled observations representative of current velocities in the upper 0.5 m of the water column^52^. The maximum horizontal range is set to 40 km, and the nominal range and angular resolutions are 1 km and 5°, respectively. This land-based network has proved to be useful in the characterization of the Atlantic jet^53^. For further details about the fundamentals and applications in the Mediterranean Sea of this consolidated remote-sensing technology, see Lorente et al.^54^.

## Supplementary Information

Supplementary Information.

## Data Availability

The original datasets generated and analyzed during the current study will be made publicly available in the Figshare repository, accessible via [https://doi.org/10.6084/m9.figshare.c.7583429.v1], upon publication. All relevant data are available from the corresponding author on reasonable request.
