# Supplementary material for: Details on the transport of European eel larvae through the Strait of Gibraltar into the Mediterranean Sea
Source: Sci Rep. 2025 Jan 6;15:1006. doi: 10.1038/s41598-024-82929-z (PMC11704197; doi:10.1038/s41598-024-82929-z)
Supplement: Supplementary file 1 — Supplementary Information. [file 41598_2024_82929_MOESM1_ESM.docx]

Supplementary table S1. Details of IKMT hauls during the survey, including station number and haul name, date and time of sampling, depth of gear, geographical coordinates (latitude and longitude), total depth, towing speed, ship’s course, wind direction and velocity, and the rope length deployed for the gear.

| **Station** | **Haulname** | **Date / Time UTC** | **Device** | **Depth of gear (m)** | **Latitude** | **Longitude** | **Sonar Depth (m)** | **Speed [kn]** | **Course [°]** | **Wind Dir**  **[°]** | **Wind Velocity [m s^-1^ ]** | **Rope Length [m]** |
| --- | --- | --- | --- | --- | --- | --- | --- | --- | --- | --- | --- | --- |
| M185_41-3 | G1 | 20.11.22 08:42 | Isaacs-Kidd Midwater Trawl | 0 | 35° 56,119' N | 005° 36,790' W | 710 | 2 | 275 | 304 | 6.40 | 0 |
| M185_41-3 |  | 20.11.22 08:54 | Isaacs-Kidd Midwater Trawl | 200 | 35° 56,119' N | 005° 37,214' W | 717 | 2 | 246 | 317 | 7.10 | 313 |
| M185_41-3 |  | 20.11.22 09:09 | Isaacs-Kidd Midwater Trawl | 20 | 35° 56,130' N | 005° 37,725' W | 706 | 2 | 277 | 290 | 6.10 | 30 |
| M185_41-3 |  | 20.11.22 09:19 | Isaacs-Kidd Midwater Trawl | 200 | 35° 56,135' N | 005° 38,123' W | 695 | 2 | 270 | 296 | 6.20 | 320 |
| M185_41-3 |  | 20.11.22 09:41 | Isaacs-Kidd Midwater Trawl | 0 | 35° 56,142' N | 005° 38,870' W | 625 | 2 | 274 | 293 | 6.00 | 0 |
|  |  |  |  |  |  |  |  |  | Mean | 300 | 6.4 |  |
| M185_41-7 | G2 | 20.11.22 16:19 | Isaacs-Kidd Midwater Trawl | 0 | 35° 56,219' N | 005° 36,808' W | 711 | 1 | 158 | 266 | 8.70 | 0 |
| M185_41-7 |  | 20.11.22 16:35 | Isaacs-Kidd Midwater Trawl | 200 | 35° 56,163' N | 005° 36,833' W | 716 | 1 | 19 | 273 | 7.90 | 392 |
| M185_41-7 |  | 20.11.22 16:54 | Isaacs-Kidd Midwater Trawl | 20 | 35° 56,118' N | 005° 37,107' W | 715 | 0 | 270 | 266 | 7.30 | 31 |
| M185_41-7 |  | 20.11.22 17:05 | Isaacs-Kidd Midwater Trawl | 200 | 35° 56,111' N | 005° 37,183' W | 717 | 1 | 243 | 282 | 8.40 | 381 |
| M185_41-7 |  | 20.11.22 17:31 | Isaacs-Kidd Midwater Trawl | 0 | 35° 56,097' N | 005° 37,433' W | 712 | 0 | 287 | 290 | 8.00 | 0 |
|  |  |  |  |  |  |  |  |  | Mean | 275.4 | 8.1 |  |
| M185_41-8 | G3 | 20.11.22 19:05 | Isaacs-Kidd Midwater Trawl | 0 | 35° 56,156' N | 005° 37,154' W | 712 | 2 | 273 | 263 | 6.50 | 0 |
| M185_41-8 |  | 20.11.22 19:18 | Isaacs-Kidd Midwater Trawl | 200 | 35° 56,132' N | 005° 37,543' W | 708 | 2 | 264 | 283 | 6.40 | 313 |
| M185_41-8 |  | 20.11.22 19:33 | Isaacs-Kidd Midwater Trawl | 20 | 35° 56,130' N | 005° 38,200' W | 681 | 2 | 261 | 287 | 7.60 | 34 |
| M185_41-8 |  | 20.11.22 19:42 | Isaacs-Kidd Midwater Trawl | 200 | 35° 56,126' N | 005° 38,651' W | 636 | 2 | 262 | 287 | 7.40 | 342 |
| M185_41-8 |  | 20.11.22 19:59 | Isaacs-Kidd Midwater Trawl | 20 | 35° 56,125' N | 005° 39,553' W | 596 | 3 | 271 | 284 | 6.20 | 34 |
| M185_41-8 |  | 20.11.22 20:05 | Isaacs-Kidd Midwater Trawl | 100 | 35° 56,122' N | 005° 39,920' W | 584 | 3 | 264 | 299 | 6.40 | 234 |
| M185_41-8 |  | 20.11.22 20:23 | Isaacs-Kidd Midwater Trawl | 0 | 35° 56,120' N | 005° 40,830' W | 522 | 2 | 262 | 290 | 5.20 | 0 |
|  |  |  |  |  |  |  |  |  | Mean | 284.7 | 6.5 |  |
| M185_41-9 | G4 | 20.11.22 21:12 | Isaacs-Kidd Midwater Trawl | 0 | 35° 56,150' N | 005° 37,350' W | 713 | 2 | 271 | 280 | 4.70 | 0 |
| M185_41-9 |  | 20.11.22 21:22 | Isaacs-Kidd Midwater Trawl | 100 | 35° 56,139' N | 005° 37,808' W | 707 | 2 | 271 | 293 | 6.70 | 176 |
| M185_41-9 |  | 20.11.22 21:30 | Isaacs-Kidd Midwater Trawl | 10 | 35° 56,137' N | 005° 38,198' W | 682 | 2 | 270 | 290 | 5.50 | 13 |
| M185_41-9 |  | 20.11.22 21:36 | Isaacs-Kidd Midwater Trawl | 100 | 35° 56,138' N | 005° 38,482' W | 648 | 2 | 277 | 298 | 6.10 | 181 |
| M185_41-9 |  | 20.11.22 21:45 | Isaacs-Kidd Midwater Trawl | 10 | 35° 56,140' N | 005° 38,912' W | 623 | 2 | 266 | 301 | 6.50 | 21 |
| M185_41-9 |  | 20.11.22 21:49 | Isaacs-Kidd Midwater Trawl | 100 | 35° 56,139' N | 005° 39,139' W | 618 | 3 | 272 | 303 | 5.70 | 158 |
| M185_41-9 |  | 20.11.22 21:57 | Isaacs-Kidd Midwater Trawl | 10 | 35° 56,141' N | 005° 39,532' W | 598 | 2 | 265 | 301 | 6.40 | 21 |
| M185_41-9 |  | 20.11.22 22:02 | Isaacs-Kidd Midwater Trawl | 100 | 35° 56,138' N | 005° 39,783' W | 588 | 2 | 271 | 299 | 6.30 | 167 |
| M185_41-9 |  | 20.11.22 22:16 | Isaacs-Kidd Midwater Trawl | 0 | 35° 56,143' N | 005° 40,503' W | 533 | 2 | 274 | 280 | 5.10 | 0 |
|  |  |  |  |  |  |  |  |  | Mean | 293.9 | 5.9 |  |
| M185_41-11 | G5 | 21.11.22 01:00 | Isaacs-Kidd Midwater Trawl | 0 | 35° 56,092' N | 005° 36,758' W | 710 | 0 | 82 | 305 | 3.10 | 0 |
| M185_41-11 |  | 21.11.22 01:12 | Isaacs-Kidd Midwater Trawl | 100 | 35° 56,102' N | 005° 36,731' W | 706 | 1 | 111 | 288 | 5.00 | 223 |
| M185_41-11 |  | 21.11.22 01:23 | Isaacs-Kidd Midwater Trawl | 30 | 35° 56,093' N | 005° 36,713' W | 704 | 0 | 124 | 278 | 5.40 | 28 |
| M185_41-11 |  | 21.11.22 01:30 | Isaacs-Kidd Midwater Trawl | 100 | 35° 56,081' N | 005° 36,725' W | 706 | 0 | 289 | 279 | 6.40 | 248 |
| M185_41-11 |  | 21.11.22 01:41 | Isaacs-Kidd Midwater Trawl | 30 | 35° 56,065' N | 005° 36,705' W | 703 | 0 | 224 | 275 | 7.10 | 25 |
| M185_41-11 |  | 21.11.22 01:46 | Isaacs-Kidd Midwater Trawl | 100 | 35° 56,064' N | 005° 36,710' W | 705 | 0 | 67 | 268 | 7.00 | 246 |
| M185_41-11 |  | 21.11.22 02:00 | Isaacs-Kidd Midwater Trawl | 30 | 35° 56,062' N | 005° 36,667' W | 701 | 0 | 189 | 276 | 6.60 | 27 |
| M185_41-11 |  | 21.11.22 02:08 | Isaacs-Kidd Midwater Trawl | 100 | 35° 56,075' N | 005° 36,692' W | 702 | 0 | 319 | 268 | 7.30 | 264 |
| M185_41-11 |  | 21.11.22 02:26 | Isaacs-Kidd Midwater Trawl | 0 | 35° 56,143' N | 005° 36,747' W | 706 | 0 | 262 | 266 | 7.30 | 0 |
|  |  |  |  |  |  |  |  |  | Mean | 278.1 | 6.1 |  |
| M185_41-12 | G6 | 21.11.22 02:41 | Isaacs-Kidd Midwater Trawl | 0 | 35° 56,107' N | 005° 36,737' W | 707 | 0 | 47 | 259 | 7.50 | 0 |
| M185_41-12 |  | 21.11.22 02:51 | Isaacs-Kidd Midwater Trawl | 100 | 35° 56,105' N | 005° 36,720' W | 707 | 0 | 122 | 266 | 6.10 | 258 |
| M185_41-12 |  | 21.11.22 03:03 | Isaacs-Kidd Midwater Trawl | 30 | 35° 56,094' N | 005° 36,709' W | 703 | 0 | 298 | 263 | 6.10 | 25 |
| M185_41-12 |  | 21.11.22 03:11 | Isaacs-Kidd Midwater Trawl | 100 | 35° 56,088' N | 005° 36,731' W | 706 | 0 | 193 | 260 | 7.30 | 264 |
| M185_41-12 |  | 21.11.22 03:24 | Isaacs-Kidd Midwater Trawl | 30 | 35° 56,075' N | 005° 36,705' W | 705 | 0 | 331 | 268 | 7.80 | 25 |
| M185_41-12 |  | 21.11.22 03:31 | Isaacs-Kidd Midwater Trawl | 100 | 35° 56,098' N | 005° 36,726' W | 705 | 0 | 64 | 279 | 7.20 | 263 |
| M185_41-12 |  | 21.11.22 03:45 | Isaacs-Kidd Midwater Trawl | 30 | 35° 56,098' N | 005° 36,723' W | 704 | 0 | 300 | 279 | 7.20 | 10 |
| M185_41-12 |  | 21.11.22 03:52 | Isaacs-Kidd Midwater Trawl | 100 | 35° 56,118' N | 005° 36,738' W | 706 | 0 | 251 | 269 | 7.30 | 255 |
| M185_41-12 |  | 21.11.22 04:11 | Isaacs-Kidd Midwater Trawl | 0 | 35° 56,173' N | 005° 36,619' W | 688 | 0 | 290 | 270 | 7.00 | 0 |
|  |  |  |  |  |  |  |  |  | Mean | 268.1 | 7.1 |  |
| M185_41-14 | G7 | 21.11.22 07:02 | Isaacs-Kidd Midwater Trawl | 0 | 35° 56,090' N | 005° 36,971' W | 716 | 1 | 262 | 233 | 9.40 | 0 |
| M185_41-14 |  | 21.11.22 07:13 | Isaacs-Kidd Midwater Trawl | 100 | 35° 56,087' N | 005° 37,266' W | 715 | 2 | 274 | 231 | 7.90 | 260 |
| M185_41-14 |  | 21.11.22 07:25 | Isaacs-Kidd Midwater Trawl | 30 | 35° 56,086' N | 005° 37,638' W | 709 | 2 | 273 | 231 | 9.40 | 24 |
| M185_41-14 |  | 21.11.22 07:33 | Isaacs-Kidd Midwater Trawl | 100 | 35° 56,081' N | 005° 37,924' W | 698 | 2 | 265 | 230 | 9.20 | 259 |
| M185_41-14 |  | 21.11.22 07:45 | Isaacs-Kidd Midwater Trawl | 30 | 35° 56,081' N | 005° 38,380' W | 655 | 2 | 270 | 226 | 8.90 | 30 |
| M185_41-14 |  | 21.11.22 07:53 | Isaacs-Kidd Midwater Trawl | 100 | 35° 56,077' N | 005° 38,720' W | 631 | 2 | 274 | 224 | 10.00 | 269 |
| M185_41-14 |  | 21.11.22 08:06 | Isaacs-Kidd Midwater Trawl | 30 | 35° 56,070' N | 005° 39,293' W | 609 | 2 | 268 | 222 | 8.90 | 25 |
| M185_41-14 |  | 21.11.22 08:14 | Isaacs-Kidd Midwater Trawl | 100 | 35° 56,065' N | 005° 39,671' W | 592 | 3 | 266 | 221 | 9.10 | 259 |
| M185_41-14 |  | 21.11.22 08:32 | Isaacs-Kidd Midwater Trawl | 0 | 35° 56,089' N | 005° 40,500' W | 539 | 2 | 296 | 216 | 9.70 | 0 |
|  |  |  |  |  |  |  |  |  | Mean | 226 | 9.2 |  |
